# Hybrid deep learning models for fake news detection: case study on Arabic and English languages

**DOI:** 10.3389/fdata.2025.1683786

**Published:** 2026-01-06

**Authors:** Baqer M. Merzah, Jafar Razmara, Zolfaghar Salmanian

**Affiliations:** Department of Computer Science, Faculty of Mathematics, Statistics, and Computer Science, University of Tabriz, Tabriz, Iran

**Keywords:** deep learning, fake news detection, multi-channel CNN, dual BiLSTM, transformers

## Abstract

**Introduction:**

Fake news has become a significant threat to public discourse due to the swift spread of online content and the difficulty of detecting and distinguishing it from real news. This challenge is further amplified by society's increasing dependence on online social networks. Many researchers have developed machine learning and deep learning models to combat the spread of misinformation and identify fake news. However, the studies focused on a single language, and the performance analysis achieved a low accuracy, especially for Arabic, which faces challenges due to resource constraints and linguistic intricacies.

**Methods:**

This paper introduces an effective deep-learning technique for fake news detection (FND) in Arabic and English. The proposed model integrates a multi-channel Convolutional Neural Network (CNN) and dual Bidirectional Long Short-Term Memory (BiLSTM), parallelly capturing semantic and local textual features embedded by a pre-trained FastText model. Subsequently, a global max-pooling layer was added to reduce dimensionality and extract salient features from the sequential output. Finally, the model classifies news as fake or real. Moreover, the model is trained and evaluated on three benchmark datasets, AFND and ANS, Arabic datasets, and WELFake, an English dataset.

**Results:**

Experimental results highlight the model's effectiveness and performance superiority over state-of-the-art (SOTA) approaches, with (94.43 ± 0.19) %, (71.63 ± 1.45) %, and (98.85 ± 0.03) %, accuracy on AFND, ANS and WELFake, respectively.

**Discussion:**

This work provides a robust approach to combating misinformation, offering practical applications in enhancing the reliability of information on social networks.

## 1 Introduction

Online social networks (OSNs) like Facebook, Twitter, and Instagram have emerged as the major sources of information in recent years. They make sharing news articles and trending topics easy. However, this ease of sharing information brought a major challenge: the credibility of that information. Fake news is fabricated or misleading information that presents itself as an actual news item and gets circulated through various channels (Lazer et al., 2018) to mislead people and influence their opinions or decisions (Giglou et al., 2020). Fake news is additionally referred to as misinformation, disinformation, hoax, and rumor in the relevant literature, all accounting for various types of false information (Nasir et al., 2021). During the 2016 U.S. presidential elections, fake news widely proliferated (Samadi et al., 2021; Alghamdi et al., 2024). During that time, the fake accounts thronged social media platforms like Twitter and Facebook and indulged in spreading adverse information about political candidates Hillary Clinton and Donald Trump.

Recent studies indicate a growing interest in FND, as researchers seek to develop effective systems to detect misleading news in multiple languages (Faustini and Covões, 2020), including English and others. This also applies to Arabic-speaking communities, especially in political, health, and celebrity news. Nonetheless, Arabic is a uniquely complex language; researchers face additional challenges when dealing with fake news in Arabic compared to other languages (Aljohani, 2024; Touahri and Mazroui, 2024). Arabic is a rich morphological language, considering its intricate grammar and multiple dialects; thus, it poses a further challenge for the natural language processing (NLP) models to perceive and analyze the Arabic text correctly. An Arabic word can change entirely due to adding prefixes and suffixes. Different contexts reflect different meanings with every word. For example, the term “ذهب” means “gold” if it comes as a noun, but could refer to “to go” when it is used as a verb. Moreover, the researchers face a shortage of resources in Arabic compared to English, which further limits the ability to develop models that could identify fake news effectively (Al Anezi, 2022; Alnabrisi and Saad, 2024). At the core of all these challenges, the unavailability of proper databases for training such models in Arabic remains a significant challenge. Therefore, due to these challenges, the domain of FND in Arabic continues to see insufficient study and unsatisfactory outcomes, presenting a substantial problem for scholars. To systematically illustrate these challenges and to highlight the specific architectural considerations required to address them, Table 1 provides a detailed breakdown of key linguistic complexities inherent in Arabic. It presents each challenge with a practical example, compares it to English to clarify the distinction for a broader audience, outlines its direct implications for FND, and introduces the fundamental architectural principle needed for its mitigation.

**Table 1:** Key linguistic challenges in Arabic FND.

**Linguistic challenge**	**Arabic sample**	**Arabic description**	**English comparison**	**Implications for FND**	**Architectural principle for mitigation**
Lexical and morphological ambiguity	ذهب.. من سيدات سويف بني محافظ النماذج من 15 يكرم الناجحة النسائية	The word ‘ذهب' (dhahab) is ambiguous. It can mean “gold” (noun), “he went” (verb), or be used metaphorically for “golden/excellent women”. The written form is identical for the noun and the metaphor.	The word “Golden” in “Golden opportunity” is a clear adjective and cannot be confused with a verb or another noun in the same form.	A model must deeply understand the context to differentiate metaphor from literal meaning. This ambiguity can be exploited in fake news with misleading headlines.	Addressing this requires analyzing the full sentence context. Therefore, a **bidirectional processing architecture** is a suitable approach, as it allows context from both past and future words to inform the meaning of an ambiguous term.
Polysemy (multiple meanings)	بوفاةتفجعماهلعين 40) ابوليل هبه الشابة (عاماً	The word ‘عين' (ayn) has numerous, unrelated meanings, including “part of a place name” (in Ayn Māhil), “eye”, “water spring”, “the thing itself”, or “the essence”, may be a verb, and means “to choose” or “to employ”	A word like “Apple” can mean a fruit or a company, but the range of meanings is far less broad and contextually distinct.	A model might incorrectly associate a place name with unrelated topics like health or natural disasters simply due to the polysemous nature of the word ‘عين‘ (ayn), leading to misclassification.	The ability to capture **long-range contextual dependencies** is essential. Architectures capable of processing entire sequences can learn to identify a word as part of a proper noun rather than an isolated, ambiguous token.
Complexity of verb forms and derived patterns	Root (ق-ت-ل) 1. ‘قَتَلَ‘ (qatala): He killed 2. ‘قاتَلَ‘ (qātala): He fought 3. ‘اقتَتَلَ‘ (iqtatala): They fought each other.	A subtle change in an internal vowel or consonant pattern (a morphological process) drastically alters the meaning from a one-sided action to a reciprocal one.	To express these meanings, completely different words are used (“He killed” vs. “He fought”), making them lexically distinct and easier to differentiate.	Propagandists can slightly alter a verb's pattern to escalate a “clash” (qātala) into a “massacre” (qattala - an intensive form), deceiving models not finely tuned to these critical morphological nuances.	To capture these deep semantic links, it is beneficial to use **sub-word-level information**. An embedding strategy that recognizes character n-grams can encode the shared root and subtle pattern changes between different words.
Agglutination (prefixes and suffixes)	A single word: “وسيكلفهم”	A single word can combine conjunctions, tense markers, a verb, and a pronoun. This example breaks down into four parts: “و” (And) + “س” (will) + “يكلف” (it costs) + “هم” (them).	The equivalent requires four separate words: “And it will cost them”.	This makes tokenization and morphological analysis both critical and difficult. A single error in segmenting a prefix or suffix can alter the entire meaning of a phrase that can be exploited in fake news.	An effective approach is to utilize **pre-trained embeddings** built on a diverse corpus that can handle complex agglutinative forms by representing them based on their constituent parts.
Inconsistent transliteration of foreign names	تغريدةفيشوارزنيغروقال	The same foreign name can be transliterated in multiple ways based on phonetics (e.g., شوارزنيجر, شوارزنيغر)	The name has a single, standard spelling: “Schwarzenegger”.	A model fails to aggregate all information related to a specific individual if their name appears in different transliterated forms. This severely hinders the ability to track the propagation of fake news or build a comprehensive profile of the individual's statements.	Utilizing **sub-word level information** in the embedding layer is an effective strategy, as it can map different transliterations of the same name to a similar vector space by recognizing shared character patterns.

Early studies in FND leaned on traditional, frequency-based methods for representing text. Aljwari et al. (2022) and Faustini and Covões (2020) used Bag-of-Words (BoW) and Term Frequency-Inverse Document Frequency (TF-IDF) to extract features. Subsequently, machine learning (ML) models were employed to detect whether news articles are real or fake. Sedik et al. (2022) and Ouassil et al. (2022) identified credible news by combining Word2Vec and GloVe word embeddings with deep learning (DL) methods, including Recurrent Neural Networks (RNNs), Convolutional Neural Networks (CNNs), and Long Short-Term Memory (LSTM) networks. Other studies, AlEsawi and Haqi Al-Tai (2024) and Samadi et al. (2021), have endeavored to integrate transformer pre-trained models, including BERT and GPT-2, with DL-based models. This transition to DL allowed models to autonomously extract more significant features from the data, resulting in progress in detecting bogus news.

Significant progress has been made in FND in English; there are very scant studies combining CNN and LSTM to evaluate the validity of news articles in Arabic. Integrated CNN and LSTM models have been found to perform differently on different datasets (Verma et al., 2021). This lacuna results from the absence of exhaustive experimentation with influential hyperparameters. The hyperparameters, like the number of LSTM layers, CNN channels, and filter dimensions, are not well studied and are mostly ineffective across varied fake news datasets of differing sizes. This lack of adaptability ends up discouraging model generalizability. The method proposed employs an ensemble of dual BiLSTM and multi-channel CNN for FND in Arabic and English to tackle these problems.

This paper introduces an effective model based on pre-trained word embedding and an ensemble of DL models for FND. The main innovation of our study is proposing a four-layered model comprising an input, a word embedding, a feature extraction, and a classification layer. At first, news articles are input into the system for the preprocessing process to filter out irrelevant information. Next, the word embedding layer takes over the responsibility for the representation of the text data as numerical vectors by using the FastText pre-trained approach. The core of the model is the feature representation layer in the form of two methodologies running in parallel: dual BiLSTM and multi-channel CNN. The BiLSTM captures sequential information and long-range dependencies in text, while the multi-channel CNN captures the local features from the text using different filter sizes (2, 3, and 4), enabling the collection of patterns of variable lengths. This allows the capture of patterns with variable lengths. The resulting representations from these layers are combined and further fed into a global max-pooling layer that is important in reducing dimensionality and critical in extracting the most outstanding characteristics from the various concatenated outputs. Ultimately, in the classification layer, the global max-pooling results are fed to dense layers to produce a final prediction: whether the input text is “Real” or “Fake”. The architecture utilizes the advantages of BiLSTM and CNN to capture many facets of textual data, improving the model's efficacy in FND while facing challenges in spotting misinformation.

Despite the progress made, a look at the present literature points to a critical gap in the literature. Many of the dominant models adopt DL ning architectures like CNNs or LSTMs, individually or sequentially, and these might limit their ability to simultaneously exploit a range of subtle textual features. Specifically, CNNs are particularly effective at detecting local features (e.g., important phrases and n-grams), while BiLSTMs are particularly effective at detecting long-distance contextual relations in sequences. The potential to combine these respective strengths in a parallel architecture is left too poorly studied, particularly in a context. This is the crux of our key contribution: we present an original hybrid design combining a Multi-Channel CNN (to extract features of mixed length) with a Dual BiLSTM (to achieve a richer contextual understanding) running in parallel. This design allows the model to simultaneously leverage the respective advantages of both approaches and construct a more robust and thorough feature representation to exploit this identified gap.

The rest of the paper is structured as follows: Section 2 examines the pertinent literature on FND. Section 3 delineates the design technique of the proposed model. Section 4 presents the experimental results, alongside substantial comparison to the SOTA results, and discusses an ablation study. Conclusion and possibilities for future enhancements are presented in Section 5.

## 2 Related work

While considerable studies exist on FND, the Arabic language has received limited attention in this area, resulting in a noticeable gap. To better understand the landscape of existing approaches, the literature review is categorized into FND for English and Arabic languages.

### 2.1 English fake news detection

Ahmed et al. (2017) suggested a model for N-gram analysis. They examined feature extraction techniques [Term Frequency (TF) and Term Frequency-Inverse Document Frequency (TF-IDF)] and six various ML approaches to online FND. Using TF-IDF and Linear Support Vector Machine (LSVM) for feature extraction and classification, attained the best accuracy. Nasir et al. (2021) investigated whether CNN-LSTM was efficient for Twitter FND. Adding a one-dimensional CNN after the word embedding in the LSTM model improved accuracy to 80%. FNDNet, introduced by Kaliyar et al. (2020), takes advantage of the GloVe pre-trained model for creating word embedding vectors. The architecture consists of three parallel CNNs with varying kernel sizes to extract multiscale information from the text. Goldani et al. (2021) presented a new method for FND, capsule neural networks (CapsNet). It is based on two architectures to learn varying lengths of news statements and non-static embedding in learning.

In 2021, Saleh et al. (2021) implemented an optimized CNN architecture in which TF-IDF was employed to perform feature extraction, after which six layers were added to extract both low and high-level features. At each level, the parameters were tuned using Hyperopt optimization techniques. A novel model for FND was adopted by Mohapatra et al. (2022) through a hybrid model consisting of a BiLSTM network alongside a self-attention mechanism. The included structure of the BiLSTM, self-attention, and other layers can pick out and classify fake news articles correctly, distinguishing them from actual ones. Khan et al. (2022) introduced BiCHAT, an innovative solution for spotting hate speech. It combines BERT embeddings with the power of BiLSTMs, deep CNNs, and hierarchical attention, making it a really interesting approach in the field. Meanwhile, Fazil et al. (2023) introduced a new approach for hate speech prediction by leveraging the combined strengths of multi-channel CNNs and BiLSTM cells with an attention mechanism.

In 2025, Jain et al. (2025) suggested a hybrid CNN-BiLSTM model enhanced by Harris Hawks Optimization (HHO) for selecting features in FND. The model includes data pre-processing, extraction of 80 linguistic features, optimization by HHO reducing redundancy, and classification by CNN and sequential dependence by BiLSTM. The model is tested using ISOT, Kaggle, ConFake, and McIntire datasets, and outperforms SOTA methods. Chen and Yin (2025) tested the performance of DistilBERT and CNN-LSTM variants using GloVe embeddings on a Kaggle dataset and reported 99.65% accuracy for DistilBERT while beating baseline models, but being highly computationally costly and restricted to the English language. Finally, Raza et al. (2025) proposed a BERT-based framework with progressive training for FND, using the WELFake dataset. Methods include BERT fine-tuning with episode-based learning for textual analysis. The model achieved 95.3% accuracy, outperforming baselines. Table 2 gives a description of the most important studies about the English FND.

**Table 2:** Summary of related studies on English FND.

**Reference**	**Approach**	**Dataset**	**Findings**	**Limitations**
Ahmed et al. (2017)	N-gram, TF-IDF, LSVM	Kaggle 12,600 real 12,600 fake	Achieved 92% accuracy using n-gram features and Linear SVM; effective for political news.	Limited to political news; small dataset
Nasir et al. (2021)	CNN-RNN	ISOT FA-KES	The proposed hybrid CNN-RNN model achieves 60% accuracy on FA-KES and 99% accuracy on ISOT, performing strongly on homogeneous data.	Poor generalization across datasets; over-fitting on small data.
Kaliyar et al. (2020)	Deep CNN (FNDNet)	Kaggle 20,800 articles	Developed FNDNet, a deep CNN achieving 98.36% accuracy without hand-crafted features; low false positive rate (0.59%).	Poor generalization, trained only on a single dataset.
Goldani et al. (2021)	Capsule Neural Network	ISOT LIAR	The proposed model achieved 99.8% and 39.5% for ISOT and LIAR, respectively. Outperformed SOTA methods.	The model finds it challenging to distinguish between nuanced labels in the LIAR dataset
Saleh et al. (2021)	Optimized CNN	Kaggle FakeNewsNet FA-KES ISOT	The OPCNN-FAKE model outperformed ML models (SVM, RF) and DL models (RNN, LSTM)	Lower performance on a smaller and imbalanced dataset
Mohapatra et al. (2022)	BiLSTM and self-attention	Fake News dataset	The suggested model outperforms DNN, LSTM, CNN, BiGRU, and CNN+BiLSTM methods, achieving 98.65% accuracy	The single and imbalanced dataset is the model's primary limitation
Khan et al. (2022)	BERT+ BiLSTM+ CNN + Attention (BiCHAT)	Three Twitter-related datasets	The model integrated the strengths of BERT, CNN, and BiLSTM and outperformed SOTA methods.	The model has not been evaluated on multilingual Twitter text
Fazil et al. (2023)	Deep CNN-BiLSTMs- Attention	Three Twitter-related datasets	The multi-channel approach effectively captures n-gram semantics at various levels. Outperformed SOTA by 10% on an imbalanced dataset	Its performance on multilingual text has not been evaluated, and it has not been compared with various transformer language models
Jain et al. (2025)	CNN-BiLSTM with HHO	ISOT Kaggle ConFake Mclntire	Novel HHO-CNN-BiLSTM, outperforming SOTA models by 98.89% in the ISOT dataset	Focus only on a single language; scalability on large datasets is untested
Chen and Yin (2025)	DistilBERT and CNN-LSTM	Kaggle 17,903 fake 20,826 real	DistilBERT achieved 99.65% accuracy, outperforming CNN-LSTM models.	High computational cost, lacks comparison with other transformers. Trained on a single dataset
Raza et al. (2025)	BERT	WELFake	BERT with progressive training achieved 95.3% outperforming baselines	Single dataset

### 2.2 Arabic fake news detection

Alzanin and Azmi (2019) presented a system for early Arabic tweet rumor detection to identify rumors before official clarifications. They used an Anti-Rumor dataset without denial cases and incorporated predefined features of sensitive content and novel features into the credibility of followers. Semi-supervised and unsupervised EM algorithms were used. The best results were obtained by using semi-supervised EM in sufficient conditions of training data. The system was limited as it depended on a private dataset and a single source. Nassif et al. (2022) developed a method based on deeply contextualized embedding for Arabic FND. In this study, they translated an English fake news dataset into Arabic. Various models were tested, such as MARBERT, Roberta, Araelectra, Arabert, GigaBert, QaribBert, ARBERT, and Arabic-BERT. The ARBERT model achieved outstanding results, attaining an accuracy of 98%, significantly better than the performance of other advanced models.

Wotaifi and Dhannoon (2023a) presented a hybrid deep neural network. They combined CNN for feature extraction and LSTM to capture long-term dependency in the textual sequences. Trained on the AraNews dataset, it outperforms Text-CNN and LSTM models individually concerning accuracy, demonstrating the effectiveness of combining these architectures for improved prediction of Arabic fake news. Fouad et al. (2022) explored various DL methods over the Arabic text for FND. The authors consider a real-life dataset, which is manually collected, along with a benchmark dataset, and combine them into one huge merged dataset to test CNN, LSTM, BiLSTM, CNN+LSTM, and CNN+BiLSTM models. Their findings showed BiLSTM achieved the highest accuracy on all the datasets, highlighting its effectiveness in FND in Arabic. Focusing on Arabic, Himdi et al. (2022) proposed an innovative approach by collecting a dataset of crowdsourced Arabic articles related to the Hajj. They utilized a custom-built NLP tool to extract textual features, namely part-of-speech tags, syntactic-semantic roles, emotional expressivity, and contextual polarity. The results obtained by various supervised ML models, comprising SVM, RF, and NB, trained on these features, reveal that RF attained an accuracy score of 78%. Wotaifi and Dhannoon (2023b) introduced an attention-based Bi-LSTM trained on the large-scale AFND dataset. Their approach, which outperforms previous models and baseline models, including Simple RNN, LSTM, and GRU, shows the efficiency of attention mechanisms for enhancing FND in Arabic.

Bahurmuz et al. (2022) developed an Arabic rumor detection model using AraBERT and MARBERT, two BERT models pre-trained on large Arabic corpora, achieving a high accuracy of up to 97% across three datasets. Their work highlights the power of transformer-based models for effectively identifying rumors in Arabic social media content. AlEsawi and Haqi Al-Tai (2024) proposed an attention mechanism-based BiLSTM model for detecting Arabic misinformation. They have used AraBERT for feature extraction. Their model has outperformed all SOTA methods on the AraNews and ArCovid19-Rumors datasets, achieving 90% and 96% accuracy, respectively. The work results concluded that including an attention mechanism and contextual embeddings enhances the performance of Arabic misinformation detection. Khalil et al. (2023) proposed an enhanced hybrid CNN-BiLSTM model for Arabic FND. Their model leveraged the concatenation of the GloVe and FastText embeddings, multiple two-dimensional convolutions, and Bi-LSTM with auxiliary output layers to make further improvements. Using the large Arabic Fake News Dataset (AFND), their model achieved 88% and 78% accuracy for binary and multi-class classification, respectively. In other research, Azzeh et al. (2024) addressed the challenge of limited Arabic data by creating a large, annotated corpus from diverse sources. They then used this corpus to test the effectiveness of pre-trained transformers like ARBERT, AraBERT, and CAMeLBERT. The results proved that the highest accuracy for text representation corresponded to CAMeLBERT and that transformer-based models were better compared to traditional ML classifiers.

In 2025, Merzah et al. (2025) suggested a hybrid DL model that integrated two BiGRU along with a self-attention mechanism and FastText word embedding for Arabic FND. The model was evaluated on the AFND dataset. Finally, Albtoush et al. (2025) evaluated ML, DL, and Arabic transformer-based models (AraBERT, AraELECTRA) for FND of Arabic headlines in AFND and ANS datasets. Transformers outperformed, with AraBERTv02 achieving 70.41% accuracy in AFND. Table 3 gives a description of the most important studies about the Arabic FND.

**Table 3:** Summary of related studies on Arabic FND.

**Reference**	**Approach**	**Dataset**	**Findings**	**Limitations**
Alzanin and Azmi (2019)	Expectation–maximization	Custom Arabic Tweets	Semi-supervised EM achieved 78.6% accuracy	Poor generalization across datasets
Nassif et al. (2022)	AraBERT AEBERT MARBERT	Custom Arabic, Translated Kaggle English	Achieved 98.8% accuracy with ARBERT on a custom dataset, outperforming priors.	Limited to political news; Arabic dialect challenges
Fouad et al. (2022)	DL models	Three Twitter datasets	BiLSTM achieved the highest accuracy (84.8% on real dataset), outperforming other models	Limited dataset size
Bahurmuz et al. (2022)	AraBERT MARBERT	Three Twitter datasets	MARBERT achieved 0.97 accuracy, outperforming baselines, and handled imbalance effectively	Arabic dialect/grammar challenges; domain-specific performance issues.
Himdi et al. (2022)	Supervised ML models	Hajj dataset 549 real 549 fake	Supervised ML (NB, RF, SVM) with textual features (emotion, linguistic, polarity, POS); Achieved 79% F1 with RF	Limited to the Hajj topic, small dataset size.
Wotaifi and Dhannoon (2023a)	Text-CNN + LSTM	AraNews 16,600 articles	Achieved 91.4% accuracy, outperforming CNN, LSTM, and prior AraNews studies	Challenges with Arabic slang and resource scarcity
Khalil et al. (2023)	CNN-BiLSTM	AFND	CNN-BiLSTM with FastText/GloVe embeddings achieved 88% binary accuracy	Single dataset, Low accuracy
Wotaifi and Dhannoon (2023b)	BiLSTM + Attention	AFND	The hybrid Bi-LSTM-attention-MLP with FastText/GloVe embeddings. The model achieved 81.73% test accuracy.	Low accuracy, single dataset
AlEsawi and Haqi Al-Tai (2024)	AraBERT-BiLSTM-attention	AraNews ArCovid19-Rumors	BiLSTM-attention achieved 0.96 accuracy on ArCovid19-Rumors and 0.90 on AraNews	Tested on only the Arabic language
Azzeh et al. (2024)	Pre-trained models AraBERT CAMELBERT	Custom (~7K Twitts)	DNN with CAMELBERT achieved 72.6% accuracy	Poor generalization across datasets
Merzah et al. (2025)	Dual BiGRU—attention	AFND	Dual BiGRU—attention with FastText embedding achieved 91.92% accuracy, outperforming SOTA methods.	Evaluated on a single dataset
Albtoush et al. (2025)	ML (SVL, LR) DL (LSTM, BiLSTM) Transformers (AraBERT, AraELECTRA)	AFND ANS	Transformers best: AraBERTv02 70.41% (AFND), AraELECTRA 77% (ANS) accuracy	Stemming inconsistencies, dialect challenges

Analysis of previous studies suggests that traditional methods like BoW and TF-IDF contain grave limitations. Traditional methods treat the text as a bag of words; hence, they only capture word frequency without semantic meaning representation. While effective independently, CNNs and BiLSTMs alone fail to capture all relevant features in text sequences. Models that did attempt to combine CNN and LSTM architectures often did so sequentially, which risks information loss between stages. Our research distinguishes itself by introducing a parallel hybrid architecture that extracts these different feature types independently before merging them. We posit that this parallel approach provides a richer, more holistic text representation, leading to improved accuracy in distinguishing fake from real news.

## 3 Materials and methods

**Figure 1:**
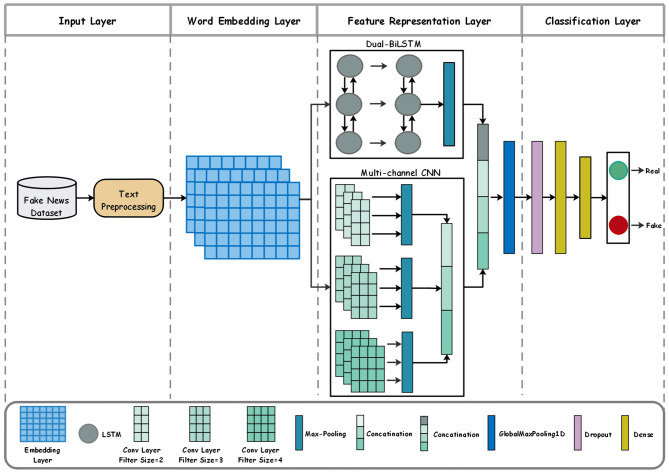
The proposed model architecture.

**Figure 2:**
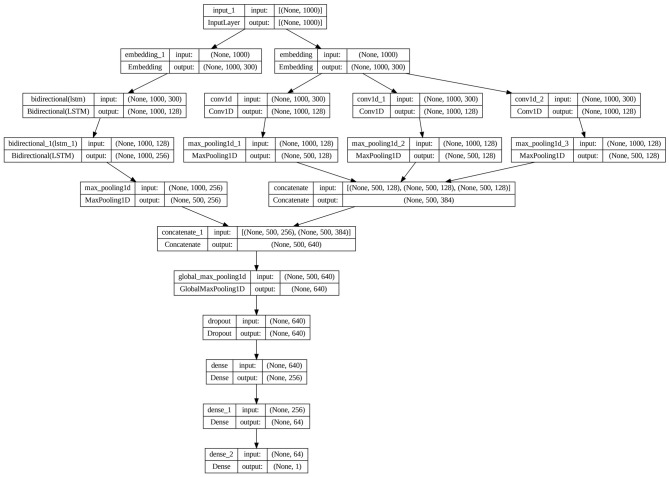
Layered architecture of the proposed model.

This section introduces a proposed model for FND in Arabic and English. Figure 1 depicts a schematic diagram of our model architecture, and Figure 2 shows the layered architecture. The proposed model includes four layers: an input layer, a word-embedding layer, a feature extraction layer, and a classification layer. The model is built upon a unified pipeline where each component is able to interact with other components in response to the issues concerning FND, especially in low-resource languages like Arabic. For example, the raw data introduces noise and linguistic variations (rich Arabic morphology and different dialects), which require special preprocessing for feeding embeddings to them and extracting significant features from those. Subsequent subsections delineate the task of each layer.

### 3.1 Datasets description

**Figure 3:**
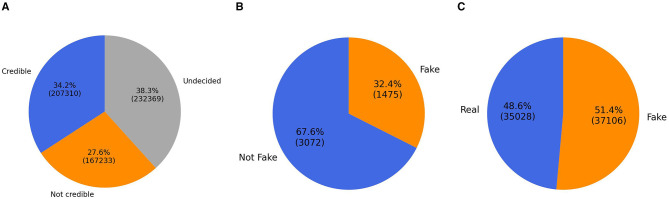
Description of the datasets. **(A)** AFND dataset. **(B)** ANS dataset. **(C)** WELFake dataset.

The model was evaluated on three benchmark datasets: two in Arabic (AFND, ANS) and one in English (WELFake). The first dataset, AFND, is a sizable Arabic dataset containing 606,912 Arabic news articles from 134 public news websites across 19 Arab countries. This dataset, provided by Khalil et al. (2022), offers a diverse representation of the Arabic news landscape. Misbar, an Arabic fact-checking platform, manually categorized these news sources into three labels: credible, not credible, or undecided. Figure 3A illustrates the count of news articles across classes. As the proposed method employs binary classification, only the credible (real) and not credible (fake) labels were used, while undecided articles were excluded.

The second dataset, the Arabic News Stance (ANS) corpus (Khouja, 2020), contains 4,547 claims created by paraphrasing or contradicting headlines from trustworthy news agencies. This dataset presented a notable class imbalance, with 3,072 “not fake” instances (67.6%) and 1,475 “fake” instances (32.4%), as shown in Figure 3B. To address this imbalance, we employed an oversampling technique. This method entails duplicating random examples from the minority class until equilibrium between the classes is achieved, thereby mitigating bias toward the majority class and enhancing the model's resilience in managing imbalanced conditions encountered in real-world applications.

Finally, WELFake (Verma et al., 2021), is an English dataset consisting of a large compilation of news data, curated with much care to be well-balanced and unbiased, and this plays a critical role in ensuring that training data is high quality and that results are delivered effectively. Although multiple free datasets can be used for FND research, most are restricted due to size, bias, and classification. Nonetheless, this limitation is addressed in the WELFake dataset as the four published datasets (McIntire, Reuters, Kaggle, and BuzzFeed) are combined, and this was for two main reasons. Firstly, these datasets have similar structures, consisting of a two-class classification: real and fake. Secondly, merging the two datasets overcomes both biases and limitations. Consequently, a WELFake dataset containing 72,134 news articles is produced, and the data can be categorized as either real or fake news: 35,028 real news articles and 37,106 fake news articles. This dataset comprises three columns: text, title, and label, with each column designated a binary label of either real or fake news. Figure 3C presents the well-balanced presence of real and fake news in the WELFake dataset.

### 3.2 Preprocessing

The datasets were cleaned and preprocessed to remove noise and irrelevant content. Preprocessing was performed on input data using multiple steps: for the Arabic dataset, these include text cleaning by removing punctuation, non-Arabic characters (Elnagar et al., 2020), and stop words, followed by tokenization and normalization using the Farasa^1^ library. However, the English dataset was preprocessed by converting text data into lowercase letters, filtering punctuation, hashtags, URLs, non-English characters, and stop words, followed by tokenization and lemmatization using the NLTK^2^ library.

### 3.3 Word embedding

Following preprocessing, the text data was transformed into the Word Embedding Layer. Word embedding techniques, like Word2Vec (Mikolov et al., 2013) and GloVe (Pennington et al., 2014), interpret the word as a single element that hampers its impact on languages with complex morphologies. The Arabic language, which is structured on roots and has complex morphology, is quite problematic for most of these approaches. FastText, developed by Facebook AI Research (Joulin et al., 2016), which was utilized in our model, resolves these limitations by using a sub-word embedding approach. FastText provides pre-trained word vectors for 157 languages, using training data from Wikipedia and Common Crawl. This approach has shown superior performance in morphologically rich languages like Arabic due to its power to encode sub-word information effectively, as it breaks words into n-grams (e.g., handling prefixes/suffixes in words like ‘كتابي' as ‘ي' + ‘كتاب'), capturing dialectal variations better than traditional embeddings like Word2Vec. FastText mitigates these issues by using a subword embedding method, where the words are decomposed into n-grams, preserving dialectal variations more effectively compared to traditional embeddings such as Word2Vec. This layer is directly connected to the feature representation layer and provides robust vectors for BiLSTM and CNN to exploit long-distance dependencies and local patterns.

### 3.4 Feature representation

The core of our proposed architecture is the feature representation layer. The proposed model processes the input article news, embedded by FastText pre-trained, using a dual BiLSTM and multi-channel CNN working in parallel to take different features out of the input text. The logical rationale for employing dual BiLSTM and multi-channel CNN in parallel stems from their complementary strengths in handling textual data, particularly for FND. Traditional sequential hybrids (e.g., CNN followed by LSTM) may lead to information loss during transitions, as local features extracted by CNN could be diluted in LSTM's sequential processing. In contrast, a parallel architecture allows independent feature extraction: BiLSTM captures bidirectional long-range dependencies and contextual nuances (essential for Arabic's morphological complexity and dialectal variations), while multi-channel CNN identifies local patterns via varying filter sizes (2, 3, 4), addressing variable-length n-grams common in both Arabic and English misinformation. The multichannel CNN and dual BiLSTM outputs are merged in the concatenation layer and then passed to a global max-pooling layer to extract salient features, mitigate overfitting, and enhance generalization.

#### 3.4.1 Dual BiLSTM

Bidirectional Long Short-Term Memory (BiLSTM) networks represent an advanced extension of the Long Short-Term Memory (LSTM) architecture. LSTM networks were first introduced to resolve the issues of classical RNNs, including vanishing gradients. LSTMs have memory cells that retain and process information over long sequences (Méndez et al., 2023). An LSTM cell consists of three gates, an input gate *i*_*t*_, a forget-gate *f*_*t*_, and an output gate *o*_*t*_, in addition to a memory cell state *C*_*t*_. The information flow in the cell is regulated to update its state based on the input using Equation 1. The amount of data to be deleted in time *t* is determined by forget-gate using Equation 2. The candidate cell value, ${\overset{\sim }{\text{C}}}_{t}$, is computed using Equation 3. Similarly, Equations 4–6 describe the calculation of the cell state *C*_*t*_, the output gate's output *o*_*t*_ and the LSTM cell's final output *h*_*t*_ at a time *t*, respectively. In these equations, *W*, **σ**, *b*, and tanh represent the weight vector, sigma function, bias vector, and hyperbolic tangent function, respectively. Meanwhile, *F*_*t*_ is the input for the BiLSTM at the timestamp *t*. Additionally, ⊗ carries out element-wise multiplication.


$$\begin{array}{l} i_{t}=\sigma \left(W_{i}\;.\left[h_{t-1},{\text{F}}_{\text{t}}\right]+{\text{b}}_{\text{i}}\right) \end{array}$$ (1)

$$\begin{array}{l} f_{t}=\sigma \left(W_{f}\;.\left[h_{t-1},{\text{F}}_{\text{t}}\right]+{\text{b}}_{\text{f}}\right) \end{array}$$ (2)

$$\begin{array}{l} {\overset{\sim }{\text{C}}}_{t}=\tanh\left(W_{\text{C}}\;.\left[{\text{h}}_{\text{t}-1},{\text{F}}_{\text{t}}\right]\right) \end{array}$$ (3)

$$\begin{array}{l} C_{t}=F_{t}⊗C_{t-1}+i_{t}⊗{\overset{\sim }{\text{C}}}_{t} \end{array}$$ (4)

$$\begin{array}{l} o_{t}=\sigma \left(W_{o}\;.\left[h_{t-1},{\text{F}}_{\text{t}}\right]+{\text{b}}_{\text{o}}\right) \end{array}$$ (5)

$$\begin{array}{l} h_{t}=o_{t}⊗\tanh\left(C_{t}\right) \end{array}$$ (6)

Bidirectional LSTMs can extract more contextual information than regular LSTMs (Tabrizchi et al., 2023). This is often important for many tasks related to NLP. BiLSTM has a pair of LSTMs, forward LSTM and backward LSTM, wherein bidirectional LSTMs extract more contextual data compared to standard LSTMs. The network utilizes both forward and backward time series to learn the current timestamp in the past and the future, which enables it to generate more accurate time-series predictions. This process leads to the formation of two hidden representations ${\vec{h}}_{t}$ and ${\overset{⃖}{h}}_{\text{t}}$ as in Equations 7, 8. Furthermore, BiLSTM computes the final representation by concatenating the data from the two LSTM networks, as demonstrated in Equation 9. Our model uses dual BiLSTM of size 64 and 128 neurons, respectively. After a pair of BiLSTMs, a max-pooling operation of pool size 2 was performed to reduce the sequence length, emphasizing key features. Finally, the proposed model transmits this encoded information to a concatenation layer for merging with the output of a multi-channel CNN.


$$\begin{array}{l} {\overset{⃖}{\text{h}}}_{\text{i}}=\text{LST}\vec{\text{M}}\left({\text{F}}_{\text{n}}\right) \end{array}$$ (7)

$$\begin{array}{l} {\vec{\text{h}}}_{\text{i}}=\text{LST}\overset{⃖}{\text{M}}\left({\text{F}}_{\text{n}}\right) \end{array}$$ (8)

$$\begin{array}{l} {\text{h}}_{\text{n}}=\left[{\overset{⃖}{\text{h}}}_{\text{i}}+{\vec{\text{h}}}_{\text{i}}\right] \end{array}$$ (9)

#### 3.4.2 Multichannel CNN

CNN is a variant of the DL model employed for image classification. However, in recent years, researchers have used it for NLP in areas such as text classification, text summarization, and question answering. Three layers make up the foundation of CNN's architecture, namely a convolution, a pooling, and a fully connected layer (Amiri et al., 2023). These layers work sequentially to extract important local features from the input. The central layer of a CNN is the convolutional layer, which excels at feature detection via matrix computation. This layer uses kernels (or filters) to operate on small chunks of the input data and introduces an activation function called ReLU (Rectified Linear Unit) to introduce non-linearity (Trueman et al., 2021). After the convolutional layer, the max pooling operation decreases the data dimensionality and highlights the most important features.

A one-dimensional convolution is applied in the current approach, as word embeddings are treated as row vectors. The model uses three parallel convolutional layers with 128 filters each and kernel sizes of (2, 3, and 4), which enables it to learn patterns of varying n-grams. Also, a max-pooling operation (pool size = 2) is performed after each convolutional layer. Finally, the output of the linked layer is merged with the output of a double BiLSTM layer for further processing.

#### 3.4.3 Global max-pooling layer

The proposed model passes the merged outputs for the multi-channel CNN and dual BiLSTM in the concatenation layer to the global max-pooling layer. This layer reduces the feature map's spatial dimensions to a fixed size, independent of the input size, but retains the most significant feature from each feature map. This will be very useful in converting variable-length sequences to fixed-size vectors, returning just one vector of 640 dimensions. The vector effectively summarizes the most salient features from the whole sequence.

The BiLSTM and multi-channel CNN are acting in parallel to make long-range context (from BiLSTM) connect with local patterns (from CNN with sizes 2, 3, 4) effectively, whereas the former prevents problems (e.g., vanishing gradient) that most RNN-type neural networks have to face, while the latter minimizes difficulty in handling variable length of patterns bearing long texts. The outputs are concatenated and refined with global max-pooling to obtain meaningful features and prevent overfitting, which is one of the limitations when training in imbalanced datasets. This interdependence leads to a more comprehensive text representation, especially in the involvement of Arabic grammar.

### 3.5 Classification layer

To tackle the problem of overfitting, a dropout layer is employed at the start of the classification layer, with a rate of 0.5. Thus, 50% of neurons are randomly eliminated throughout the training process. The first dense layer fed from the dropout layer reduces the layer's output to 256 features. This layer learns to combine the features extracted from previous layers. The second dense layer reduces the dimensionality further to 64 features, refining the learned features. Finally, the output layer outputs a single value, which is typically passed through a sigmoid activation function for binary classification tasks, providing a distribution over the two classes, “Real” and “Fake”, to give the final classification decision.

### 3.6 Experimental and hyperparameter settings

To evaluate and analyze the proposed model's efficacy and account for class imbalance, we employed stratified 5-fold cross-validation (CV). We executed all experiments using Python on Google Colab. The DL models were implemented using the TensorFlow and Keras libraries. Libraries such as Pandas, NLTK, and Farasa were used for preprocessing. The two-layer stacked BiLSTM network used in the model contains 64 and 128 memory cells, respectively, and then applies max-pooling of size 2. The multi-channel CNN has three layers, each with 128 filters, sized 2, 3, and 4, respectively. A max-pooling operation with a pool size of 2 is performed. To prevent overfitting, a dropout rate of 0.5 was used. The Adam optimizer was used with a batch size of 32 across all CV runs. Table 4 summarizes all hyperparameter values used for the experimental evaluation. Building on the stratified 5-fold CV setup, we reproduced strong transformer baselines to ensure fair comparison under identical preprocessing and data splits. For the Arabic AFND and ANS datasets, AraBERT and MARBERT were implemented using the Hugging Face Transformers library, the same tokenization, batch size of 32, and Adam optimizer with early stopping based on F1-score. For the English WELFake dataset, BERT and DistilBERT were similarly reproduced with equivalent settings.

**Table 4:** Hyperparameters used in the proposed model.

**Hyperparameter**	**Value**
Embedding dimension	300
Maximum sequence length	1,000
CNN filter size	2,3 and 4
Number of CNN filters	128
Pooling size	2
Number of neurons in BiLSTM	64 and 128
Dropout	0.5
Dense layer	256 and 64
Batch size	32
Output activation	Sigmoid
Optimization algorithm	Adam (0.001)

### 3.7 Performance evaluation metrics

To measure the effectiveness of the proposed model, four standard metrics were applied: recall (Rc), accuracy (Ac), F1-score (F1), and precision (Pr). These metrics are commonly used for FND and are calculated based on the confusion matrix components: True-Positive (TP), True-Negative (TN), False-Positive (FP), and False-Negative (FN). Pr indicates the percentage of correctly classified fake news articles to the total number of articles classified as fake news shown in Equation 10. Rc, however, represents the percentage of correctly classified fake news from the total number of fake news labels, as defined in Equation 11. F1, also called the F-measure, is the harmonic mean of precision and recall, as shown in Equation 12. Finally, Ac denotes the proportion of accurately identified real or fake news relative to the total labeled articles in the dataset, the mathematical formulation of which can be seen in Equation 13.


$$\begin{array}{l} Pr=\frac{TP}{TP+FP} \end{array}$$ (10)

$$\begin{array}{l} Rc=\frac{TP}{TP+FN} \end{array}$$ (11)

$$\begin{array}{l} F1=\frac{2\times Pr\times Rc}{\mathrm{Pr}+Rc} \end{array}$$ (12)

$$\begin{array}{l} Ac=\frac{TP+TN}{\#news} \end{array}$$ (13)

Additionally, to address class imbalance sensitivity, we computed macro-F1, which is the unweighted average of F1 across all classes, treating each class equally regardless of size. For binary classification, macro-F1 is calculated as:


$$\begin{array}{l} Marco-F1=\frac{{F1}_{Positive}+{F1}_{negative}}{2} \end{array}$$

We also report the Area Under the Curve-Precision-Recall (AUC-PR), which evaluates the trade-off between precision and recall across various thresholds.

## 4 Results

This section reports the quantitative results of the experiments carried out to assess the suggested model.

### 4.1 Model performance on benchmark datasets

**Figure 4:**
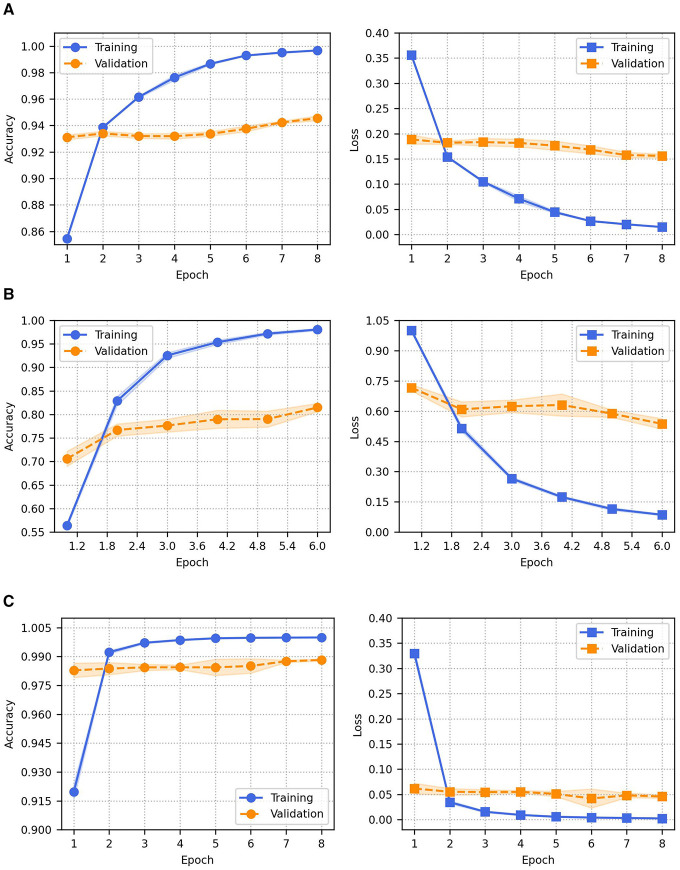
Training and validation accuracy and loss curves. **(A)** AFND dataset. **(B)** ANS dataset. **(C)** WELFake dataset.

The proposed model was trained and evaluated using AFND, ANS, and WELFake datasets. For the Arabic AFND dataset, the proposed method achieved a mean Ac of (94.43 ± 0.19) %, Pr of (95.4 ± 0.2) %, Rc of (94.5 ± 0.2) %, and F1 of (94.95 ± 0.2). For the ANS dataset, our model achieved a mean Ac of (71.63 ± 1.45) %, Pr of (77.42 ± 1.32) %, Rc of (81.9 ± 0.91) %, and F1 of (79.6 ± 1.42) %. On the English WELFake dataset, the model demonstrated superior performance with mean Ac of (98.85 ± 0.03) %, Pr of (98.8 ± 0.19) %, Rc of (98.84 ± 0.16) %, F1 of (98.82 ± 0.03). Training and validation accuracy and loss curves (averaged across stratified 5-fold CV runs) on the AFND, ANS, and WELFake datasets are shown in Figures 4A–C, respectively.

To ensure a fair and comprehensive comparison, we reproduced several strong transformer baselines: AraBERT and MARBERT for Arabic, and BERT and DistilBERT for English. All the baselines were executed in our experiment framework using the same preprocessing, splitting schemes for the data, and evaluation for metrics in order to achieve a comparable and easy-to-follow comparison to our proposed model.

### 4.2 Comparison with state-of-the-art methods

To evaluate the efficiency of our proposed model, we carried out a double comparison. Initially, we compared our work against existing SOTA methods. Next, for a fair and clear comparison, we also implemented some SOTA transformer baselines (BERT and DistilBERT for English, while AraBERT and MARBERT for Arabic) and subjected them to testing in our precise experimental setup using the same protocol of stratified cross-validation.

For the AFND dataset, our model compares with the SOTA methods: Capsule Network (Khalil et al., 2021), BiLSTM-Attention (Wotaifi and Dhannoon, 2023b), CNN-BiLSTM (Khalil et al., 2023), Ensemble (Fares et al., 2024), LSTM (Abd Elminaam et al., 2023), WLT-araBERT+BiLSTM (Turki et al., 2025). As indicated in Table 5, the performance of our model was comparatively evaluated against prominent methodologies using the AFND dataset. Our method achieved a mean Ac of (94.43 ± 0.19) %, Pr of (95.4 ± 0.2) %, Rc of (94.5 ± 0.2) %, and F1 of (94.95 ± 0.2) %. While the models by Abd Elminaam et al. (2023) and Khalil et al. (2023) were strong competitors, our model surpassed their reported Ac by 4.29% and 6.94%, respectively. Furthermore, our model showed a significant improvement of 7.95% in F1 over the CNN-BiLSTM method (Khalil et al., 2023). When compared to our reproduced baselines, our model also demonstrated superior performance, exceeding the accuracy of AraBERTv2 by 1.07% and MARBERT by 2.18%.

**Table 5:** Comparison of the proposed model against baselines and SOTA methods on the AFND dataset.

**Methods**	**Ac (%)**	**Pr (%)**	**Rc (%)**	**F1 (%)**	**Macro F1 (%)**	**AUC-PR (%)**
Capsule network (Khalil et al., 2021)	78.3	-	-	-	-	-
BiLSTM-Attention (Wotaifi and Dhannoon, 2023b)	81.73	-	-	-	-	-
CNN-BiLSTM (Khalil et al., 2023)	87.49	88	87.32	87	-	-
Ensemble (Fares et al., 2024)	89	-	-	-	-	-
WLT-araBERT+BiLSTM (Turki et al., 2025)	89.91	-	-	-	-	-
LSTM (Abd Elminaam et al., 2023)	90.14	-	-	-	-	-
AraBERTv2	93.36 ± 0.35	94.04 ± 0.46	94.11 ± 0.27	94.07 ± 0.31	93.36 ± 0.33	**98.96** **±0.91**
MARBERT	92.25 ± 1.91	93.35 ± 1.66	88.89 ± 1.43	91.07 ± 1.88	90.28 ± 1.82	97.99 ± 1.24
Proposed model	**94.43** **±0.19**	**95.4** **±0.2**	**94.5** **±0.2**	**94.95** **±0.2**	**94.31** **±0.2**	98.8 ± 0.1

The bold values indicate the highest performance metrics achieved in each comparison.

In the challenging case of the ANS dataset, our proposed approach is more effective on imbalanced data. Our model was trained on the augmented balanced data with random over-sampling techniques, which avoids the bias toward the majority class. Nevertheless, all reported performances are evaluated on the original imbalanced test set to mimic a realistic use case. This strategy proved highly effective.

Our model compares with the SOTA methods on the ANS dataset: AraGPT2 (Al-Yahya et al., 2021), BERT (Khouja, 2020), LSTM-CNN (Sorour and Abdelkader, 2022), JointBERT (Shishah, 2022), and APBTM (Abdelhakim Othman et al., 2024). As can be seen in Table 6, our hybrid model demonstrated an average Ac of (71.63 ± 1.45) % and an F1 of (79.6 ± 1.42) %. This is a great improvement over Previous SOTA for DL, such as the APBTM (Abdelhakim Othman et al., 2024) model (Ac 71.42%). We obtained SOTAs on all individual datasets used with directly comparable transformer baselines, AraBERTv2 and MARBERT, by 0.99% and 4.9% respectively. The model's high AUC -PR score of 81.85% deserves a special mention, since this seems to show that together, the blended hybrid architecture and oversampling training strategy yield a resilient model capable of dealing with drastic class imbalance.

**Table 6:** Comparison of the proposed model against baselines and SOTA methods on the ANS dataset.

**Methods**	**Ac (%)**	**Pr (%)**	**Rc (%)**	**F1 (%)**	**Macro F1 (%)**	**AUC-PR (%)**
AraGPT2 (Al-Yahya et al., 2021)	64.23	27.81	19.1	20.39	-	-
BERT Khouja, (2020)	-	64	65	64	-	-
LSTM-CNN Sorour and Abdelkader, (2022)	67	69	67	68	-	-
JointBERT (Shishah, 2022)	66	62	60	66	-	-
APBTM (Abdelhakim Othman et al., 2024)	71.42	75.6	52.38	61.88	-	-
AraBERTv2	70.64 ± 0.99	76.93 ± 1.15	80.76 ± 1.53	78.80 ± 1.05	65.47 ± 1.42	**81.98** **±1.3**
MARBERT	66.73 ± 1.6	72.87 ± 1.1	80.86 ± 2.1	76.65 ± 1.79	64.12 ± 1.6	80.32 ± 1.75
Proposed model	**71.63** **±1.45**	**77.42** **±1.32**	**81.9** **±0.91**	79.6 ± 1.42	**65.74** **±1.86**	81.85 ± 1.4

The bold values indicate the highest performance metrics achieved in each comparison.

**Figure 5:**
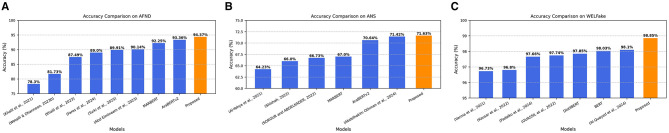
A graphical comparison of the accuracy of the proposed method with SOTA. **(A)** AFND dataset. **(B)** ANS dataset. **(C)** WELFake dataset.

Moreover, the SOTA models compared to our model, based on the WELFake dataset, are: SVM (Verma et al., 2021), N-Gram with TF-IDF and BERT (Kausar et al., 2022), Attention-based BiLSTM (Padalko et al., 2024), CNN-BiLSTM (Ouassil et al., 2022), and BERT+BiLSTM (Al-Quayed et al., 2024). Table 7 shows that the proposed model performs impressively on the WELFake dataset, beating all the previous models on key metrics. Our model obtains the highest mean Ac of (98.85 ± 0.03) %, beating BERT+BiLSTM (Al-Quayed et al., 2024) and CNN-BiLSTM (Ouassil et al., 2022), which attained an Ac of 98.1% and 97.74%, respectively. Our model also consistently outperformed our reproduced transformer baselines when directly compared. It outperformed BERT and DistilBERT with an increasing Ac of 0.82% and 1.0%, respectively. Despite small performance margins on this benchmark English dataset, the model's wide margin of victory and extremely high AUC-PR value of 99.9%, combined with its minuscule standard deviation (±0.03 for Ac), reinforce our claim that it is robust and dependable and capable of successfully identifying fake news in the context of English. A graphical comparison of the accuracy of the proposed model and SOTA methods on datasets AFND, ANS, and WELFake is shown in Figure 5.

**Table 7:** Comparison of the proposed model against baselines and SOTA methods on the WELFake dataset.

**Methods**	**Ac (%)**	**Pr (%)**	**Rc (%)**	**F1 (%)**	**Macro F1 (%)**	**AUC-PR (%)**
SVM (Verma et al., 2021)	96.73	94.6	98.61	96.56	-	-
N-Gram with TF-IDF and BERT (Kausar et al., 2022)	96.8	96.5	97	96.3	-	-
Attention-based BiLSTM (Padalko et al., 2024)	97.66	97.70	97.67	97.62	-	-
CNN-BiLSTM (Ouassil et al., 2022)	97.74	98.16	97.35	97.75	-	-
BERT+BiLSTM (Al-Quayed et al., 2024)	98.1	-	-	98.2	-	-
DistilBERT	97.85 ± 0.41	97.23 ± 0.66	98.63 ± 0.26	97.93 ± 0.39	97.85 ± 0.41	99.78 ± 0.08
BERT	98.03 ± 0.2	97.77 ± 0.45	98.42 ± 0.16	98.09 ± 0.19	98.03 ± 0.2	99.81 ± 0.04
Proposed model	**98.85** **±0.03**	**98.8** **±0.19**	**98.84** **±0.14**	**98.82** **±0.03**	**98.85** **±0.03**	**99.9** **±0.01**

The bold values indicate the highest performance metrics achieved in each comparison.

### 4.3 Ablation study

An ablation study was performed by systematically removing and adding some components to assess the efficacy of the proposed architecture. The proposed DL model incorporates three neural network components: a deep CNN (3 layers), dual BiLSTM, and a global max-pooling layer. To understand the impact of each component, we performed an ablation analysis that involved removing one element at a time: the deep CNN, the stacked BiLSTM, and the global max-pooling layer. We also evaluated a model using a single CNN and a single BiLSTM. The models are:

- **Model 1 (our model without dual BiLSTM):** In this model, the dual BiLSTM, which is responsible for capturing semantic features and contextual information, was removed from our model to evaluate the impact of its removal on the model.
- **Model 2 (our model without multi-CNN):** The multi-CNN networks, which effectively explore local features by using multiple filter sizes that enable the network to detect patterns of varying lengths, were removed.
- **Model 3 (our model without global max-pooling):** The global max-pooling layer, responsible for dimensionality reduction and extracting prominent features, is replaced with a flattened layer to convert the features to a vector for passing to the dense layer.
- **Model 4 (single BiLSTM and single CNN):** Instead of dual BiLSTM and multichannel CNN, a single BiLSTM with 128 neurons and a single CNN with 128 filters of size three were used to compare performance with the proposed model.

Table 8 presents an ablation study analyzing the impact of each model component on performance across the AFND, ANS, and WELFake datasets.

**Table 8:** Ablation analysis on the AFND, ANS, and WELFake datasets.

**Dataset**	**Methods**	**Ac (%)**	**Pr (%)**	**Rc (%)**	**F1 (%)**	**Macro F1 (%)**	**AUC-PR (%)**
AFND	Model 1	91.68 ± 0.3	92.71± 0.3	92.27 ± 0.3	92.29 ± 0.3	91.27 ± 0.54	97.43 ± 0.2
	Model 2	92.77 ± 0.43	93.23 ± 0.4	93.58 ± 0.4	93.42 ± 0.4	93.45 ± 0.4	96.42 ± 0.2
	Model 3	92.46 ± 0.2	93.32 ± 0.2	92.76 ± 0.2	93.09 ± 0.2	93.1 ± 0.28	97.66 ± 0.2
	Model 4	92.74 ± 0.25	93.28 ± 0.27	93.68 ± 0.25	93.41 ± 0.25	93.36 ± 0.18	98.46 ± 0.2
	Proposed model	**94.43** **±0.19**	**95.4** **±0.2**	**94.5** **±0.2**	**94.95** **±0.2**	**94.31** **±0.2**	**98.8** **±0.1**
ANS	Model 1	64.68 ± 2.93	71.09 ± 1.67	78.15 ± 1.57	75.34 ± 1.64	58.55 ± 2.14	70.23± 1.8
	Model 2	66.57 ± 1.6	72.86 ± 1.25	80.01 ± 1.49	76.76 ± 1.2	60.46 ± 1.67	73.08 ± 1.58
	Model 3	66.44 ± 1.42	72.74 ± 1.68	80.96 ± 1.98	76.63 ± 1.66	60.68 ± 1.49	74.65 ± 1.73
	Model 4	66.73 ± 1.78	72.87 ± 1.29	80.86 ± 4.71	76.65 ± 1.37	61.61 ± 1.78	75.34 ± 1.78
	Proposed model	**71.63** **±1.45**	**77.42** **±1.32**	**81.9** **±0.91**	**79.6** **±1.42**	**65.74** **±1.86**	**81.85** **±1.4**
WELFake	Model 1	97.51 ± 0.2	97.8 ± 0.19	97.25 ± 0.2	97.51 ± 0.18	97.33 ± 0.74	98.83 ± 0.2
	Model 2	97.79 ± 0.31	97.58 ± 0.28	98.2 ± 0.28	97.86 ± 0.3	97.61 ± 0.8	97.1 ± 0.2
	Model 3	97.99 ± 0.18	97.44 ± 0.2	98.71 ± 0.2	98 ± 0.18	97.88 ± 0.68	99.1 ± 0.2
	Model 4	98.1 ± 0.09	97.69 ± 0.2	98.7 ± 0.2	98.14 ± 0.1	98.15 ± 0.43	99.5 ± 0.2
	Proposed model	**98.85** **±0.03**	**98.8** **±0.19**	**98.84** **±0.14**	**98.82** **±0.03**	**98.85** **±0.03**	**99.9** **±0.01**

The bold values indicate the highest performance metrics achieved in each comparison.

The complete model achieves the highest performance on the AFND dataset, with a mean Ac of (94.43 ± 0.19) % and an F1 of (94.95 ± 0.2) %. Excluding the BiLSTM layer significantly impacts the results, reducing Ac to (91.68 ± 0.3) % and F1 to (92.29 ± 0.3) %. In contrast, removing the multi-CNN network results in a performance decrease, with Ac dropping to (92.77 ± 0.43) % and F1 to (92.29 ± 0.3) %. Furthermore, the effect of the global max-pooling layer is revealed when its removal decreases Ac and F1 to (92.46 ± 0.2) % and (93.09 ± 0.2) %, respectively. Finally, the configuration using a single CNN and BiLSTM record decreases Ac and F1 by (92.74 ± 0.25) % and (93.41 ± 0.25), respectively.

On the ANS dataset, our complete model again achieved the highest performance with a mean Ac of (71.63 ± 1.45) %, outperforming the four ablation configurations (Models 1–4) by margins of 4.9%, 5.19%, 5.06%, and 6.95%, respectively.

A similar study on the WELFake dataset confirms that the proposed model outperforms all other configurations, achieving a mean Ac of (98.85 ± 0.03) % and an F1 of (98.82 ± 0.03) %. In the ablated models (Models 1–4), the accuracy decreased to (97.51 ± 0.2) %, (97.79 ± 0.31) %, (97.99 ± 0.18) %, and (98.1 ± 0.09) %, respectively.

### 4.4 Error analysis

**Figure 6:**
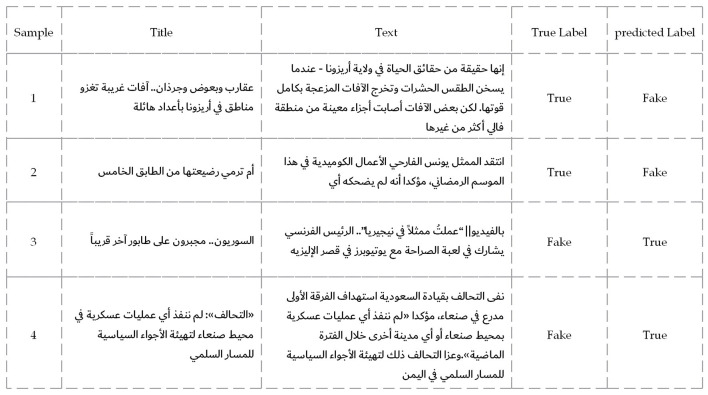
Sample news articles from the AFND dataset where the proposed model misclassified the content.

To surface concrete failure modes without relying on aggregate numbers, we qualitatively traced each misclassification in AFND, ANS, and WELFake to the textual cues most likely responsible for the model's decision. In AFND (Figure 6), the classifier overweighted sensational morphology and authority framing. The true headline “أريزونا تغزو وبعوض عقارب” (Scorpions and mosquitoes invade Arizona) was judged Fake; when the verb is neutralized (“تنتشر” instead of “تغزو”), the same content is classified correctly, indicating a causal dependence on aggressive morphology rather than the corroborating details in the body. Conversely, the fake denial “≪عملياتأيننفذلم: ≪التحالف…” (The coalition: We did not conduct any operations…) was accepted as True: the formal register and institutional attribution acted as sufficient signals despite the absence of dates, places, or external sources.

**Figure 7:**
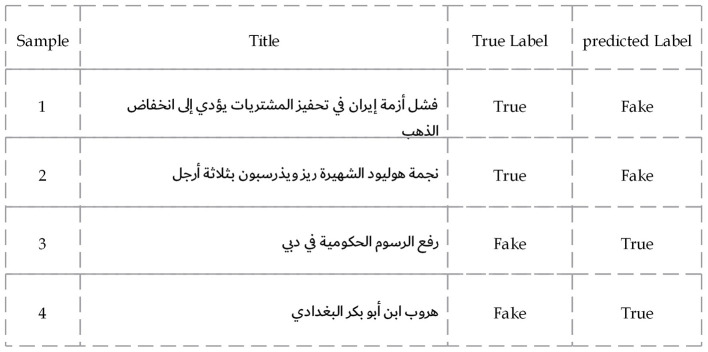
Sample news articles from the ANS dataset where the proposed model misclassified the content.

In ANS (Figure 7), two mechanisms recur: domain/locale under-coverage and a prior for bureaucratic phrasing. The true economic headline about gold prices (“الذهب انخفاض إلى يؤدي … فشل” – Failure … leads to a decline in gold) was labeled Fake even though the body encodes canonical economic causality (“السعر انخفاض → التحفيزفشل”) and contains no rumor markers; this pattern does not appear on comparable English economic items, pointing to sparse representation of Arabic financial news rather than lexical ambiguity. By contrast, plainly phrased policy claims such as “دبي في الحكومية الرسوم رفع” (Raising government fees in Dubai) were granted True despite lacking citations or quotations, revealing a stylistic prior that equates administrative tone with credibility.

**Figure 8:**
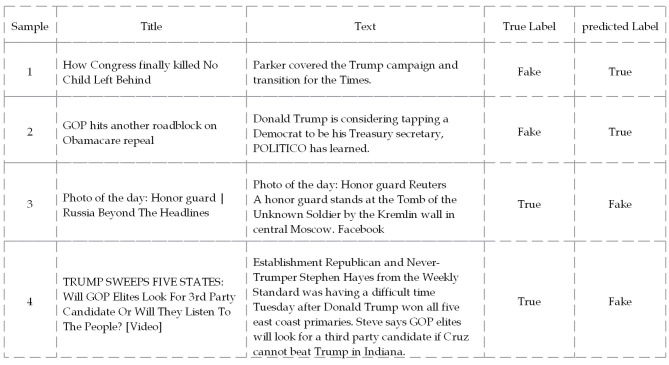
Sample news articles from the WELFake dataset where the proposed model misclassified the content.

In WELFake (Figure 8), errors reflect missing title–body entailment and sensitivity to stylistic packaging. The fake headline “How Congress finally killed No Child Left Behind” was predicted True while its body pivots to unrelated campaign coverage; a simple entailment probe—presence of the bill, legislative actors, or a repeal timeline—would yield a non-support signal. Mentions of reputable outlets also misled the classifier: “GOP hits another roadblock on Obamacare repeal” was judged True likely due to the “POLITICO has learned” cue, whereas all-caps and bracketed media tokens in similar items are down-weighted as stylistic noise. These cases jointly isolate key failure modes—aggressive-morphology priors, authority-framing bias, missing title–body entailment, and an Arabic domain-coverage gap—explaining the mistakes without aggregate statistics.

## 5 Discussion

This section provides an interpretation and analysis of the results presented above. The comparative results evidently show the superiority of our proposed hybrid DL model over the current state-of-the-art methods on both Arabic and English datasets for FND. Interestingly, the model surpassed transformer-based and ensemble approaches, demonstrating its strength and flexibility. The considerable gain in Pr, Rc, and F1, particularly on the AFND dataset, indicates the model's ability to generalize well over varied linguistic structures and news domains. Our suggested model clearly outperformed the transformer-based hybrid approach, WLT-araBERT+BiLSTM suggested by Turki et al. (2025). On a small sample of 30,000 records, their model obtained an accuracy of 89.91% under rigorous preprocessing conditions. Although their study's highest reported accuracy was 93.83%, it's important to remember that this outcome was achieved by employing only punctuation mark removal. This distinction demonstrates our model's strong performance, particularly its superiority in deep data cleaning scenarios, where it outperforms contemporary transformer-based models. This performance improvement is due to the synergistic combination of multi-channel CNNs and dual BiLSTM layers, allowing the model to effectively learn both local textual patterns and long-range contextual dependencies. The application of global max-pooling is a further strength of the model in extracting the most salient features, while the use of pre-trained FastText embeddings offers strong word representations, with special benefits for morphologically rich and low-resource languages such as Arabic.

The adoption of stratified 5-fold CV provides a more reliable performance estimate, with low standard deviations (e.g., ±0.19% for Ac on AFND) indicating model stability across data splits. The high AUC-PR scores (e.g., 98.8 ± 0.01 on AFND) and macro-F1 (94.31 ± 0.2) % confirm the model's effectiveness in imbalanced scenarios, where precision-recall trade-offs and equal class treatment are critical for minimizing false positives in fake news detection. The reproduction of transformer baselines under identical conditions further validates the proposed model's superiority, with consistent outperformance across metrics like AUC-PR and macro-F1. For instance, on AFND, the model achieves a higher Ac (94.43 ± 0.19) % than AraBERT (93.36 ± 0.35) %, underscoring the hybrid DL approach's robustness in handling class imbalance, paving the way for future cross-lingual extensions.

The ablation study conducted in the current research provides important insight into the contribution of each element in the proposed model framework for false news detection on both AFND (Arabic) and WELFake (English) datasets. Removing the dual BiLSTM layer (Model 1) resulted in the strongest decrease in accuracy and F1-score on both datasets. This finding highlights the importance of the role played by the BiLSTM in identifying sequential dependencies and contextual information in news reports. The ability of the BiLSTM to encode long-range relations in text is particularly important in detecting subtle cues distinguishing real from fake news, especially in morphologically rich languages like Arabic.

Likewise, the removal of the multi-channel CNN (Model 2) resulted in an apparent drop in performance. This highlights the value of having multiple convolutional filters in order to capture varied local features and patterns of different lengths in the text. The multi-CNN framework allows the model to identify a broad variety of linguistic and stylistic features that are typically characteristic of fake news. The deletion of the global max-pooling layer (Model 3) also led to degraded performance, signifying its usefulness in extracting the most important features from the concatenated outputs of the CNN and BiLSTM layers. It assists in dimensionality reduction while retaining the most informative parts of the feature maps.

In addition, the utilization of just one CNN and one BiLSTM (Model 4), as opposed to the stacked and multi-channel strategy, led to worse performance. This indicates that the combination of multiple channels and stacked layers offers a dramatic improvement and enables the model to learn local as well as global textual patterns more efficiently. Each component, as demonstrated by its corresponding ablation model, contributes uniquely to the model's ability to generalize across different languages and datasets. The findings accentuate the need for a balanced and carefully designed architecture, rather than any single approach, to address the complex problem of fake news detection.

A deeper analysis of the misclassified samples reveals that the errors extend beyond general linguistic complexity. For example, a large percentage of false negatives (real news labeled as fake) happened when real news articles used sensationalist or “clickbait-style” headlines (e.g., “أريزونا تغزو غريبة آفات”). The model appears to have over-indexed on this stylistic feature, penalizing legitimate news for adopting a tone commonly associated with fake news. Conversely, many false positives (fake news classified as real) were observed when the fake article successfully mimicked a formal, official tone (e.g., “عسكرية بعمليات قيامه التحالف نفي”). In these cases, the model was deceived by the professional-sounding language, demonstrating a vulnerability to sophisticated fakes that lack typical stylistic red flags. This suggests the model relies more on stylistic heuristics than on a deeper semantic analysis of the content.

The low false positive and false negative rates indicate a somewhat balanced model. The false positives often occurred due to a complete semantic disconnect between the title and the article's body, a tactic used to generate plausible-sounding but incoherent fake news. The model failed to detect this logical mismatch, especially when the text mentioned a credible source like “POLITICO,” which appeared to give the content a false sense of authenticity. Furthermore, the false negatives were frequently caused by stylistic choices in real news headlines, such as the use of rhetorical questions or a blog-like tone (e.g., “TRUMP SWEEPS FIVE STATES...”). This confirms a cross-lingual weakness: the model is overly sensitive to stylistic cues across both Arabic and English, sometimes at the expense of factual content.

In summary, though dataset imbalance is a factor, our error analysis suggests that the model's biggest weakness is its dependence on stylistic and structural heuristics instead of semantic coherence and factuality. Future research should concentrate on training the model to be more resilient against sensationalist language, to better identify logical discrepancies between titles and content, and to achieve a more complex understanding of source credibility than just keyword recognition in order to reduce these misclassifications.

## 6 Conclusion

This work uses an effective DL model for FND on both English (a high-resource language) and Arabic (a low-resource language). To create the input data, pre-trained FastText word representation is applied to produce word vectors. This generated matrix flows parallel to a multi-channel CNN and dual BiLSTM to capture semantic features and local patterns. Our model was evaluated on three benchmark datasets, achieving superior performance, as evidenced by accuracies of (94.43 ± 0.19) % on AFND, (71.63 ± 1.45) % on ANS, and (98.85 ± 0.03) % on WELFake, outperforming SOTA methods by up to 4.29%,0.21%, and 0.75%, respectively. However, our hybrid DL model achieves superior performance compared to the transformer baselines reproduced under the same stratified 5-fold CV framework. Specifically, it outperforms AraBERTv2 by 0.88% in F1 on AFND, and 0.8% in F1 on ANS, and BERT by 0.82% in F1 on WELFake. These results, including enhanced macro-F1 and AUC-PR, underscore the model's robustness in FND and its potential for real-world applications. Additionally, an ablation study was conducted to examine the effects of the components on the structure of the suggested model. Ablation studies further support our model's superiority, with the full model improving F1-scores by 1.47% on AFND, 2.84% on ANS, and 0.74% on WELFake, highlighting the hybrid architecture's effectiveness in addressing bilingual challenges. Although the proposed model has shown a promising performance, it must be acknowledged that there are some limitations and challenges. One of these challenges is the intricacy of the Arabic language, including its rich morphology, dialect diversity, and precise grammar. While FastText pre-trained word embedding addresses some of these challenges, it remains insufficient to handle linguistic details such as dialect variation. Another limitation is that the efficacy of the model was tested on just two languages, Arabic and English. Furthermore, the model's focus on textual data alone is a limitation, as real-world fake news often includes multimodal content, such as images and videos, which the current model does not consider. Lastly, the model faces difficulties in identifying nuanced forms of fake news, such as satire or sarcasm, which share linguistic features with genuine news and require deeper contextual understanding. In the future, we plan to expand our model to work with multiple languages. We will evaluate it on datasets for low-resource languages like Persian or Turkish, utilizing techniques such as transfer learning, which is a promising research direction. Additionally, we aim to extend our model to a multimodal framework, incorporating images and videos using approaches like vision transformers. This will address the limitations of text-only detection in real-world misinformation scenarios. The suggested model can be applied by OSN service providers for FND, demonstrating its efficacy on real-world datasets.

## Data Availability

Publicly available datasets were analyzed in this study. This data can be found here: The AFND dataset can be found at: https://data.mendeley.com/datasets/67mhx6hhzd/1. The ANS dataset can be found at: https://github.com/latynt/ans. The WELFake dataset can be found at: https://zenodo.org/records/4561253.

## Footnotes

1
https://farasa.qcri.org/

2
https://www.nltk.org/
